# *Edwardsiella* Comparative Phylogenomics Reveal the New Intra/Inter-Species Taxonomic Relationships, Virulence Evolution and Niche Adaptation Mechanisms

**DOI:** 10.1371/journal.pone.0036987

**Published:** 2012-05-10

**Authors:** Minjun Yang, Yuanzhi Lv, Jingfan Xiao, Haizhen Wu, Huajun Zheng, Qin Liu, Yuanxing Zhang, Qiyao Wang

**Affiliations:** 1 State Key Laboratory of Bioreactor Engineering, East China University of Science and Technology, Shanghai, People's Republic of China; 2 Shanghai – MOST Key Laboratory of Health and Disease Genomics, Chinese National Human Genome Center at Shanghai, Shanghai, China; University of Edinburgh, United Kingdom

## Abstract

*Edwardsiella* bacteria are leading fish pathogens causing huge losses to aquaculture industries worldwide. *E. tarda* is a broad-host range pathogen that infects more than 20 species of fish and other animals including humans while *E. ictaluri* is host-adapted to channel catfish causing enteric septicemia of catfish (ESC). Thus, these two species consist of a useful comparative system for studying the intricacies of pathogen evolution. Here we present for the first time the phylogenomic comparisons of 8 genomes of *E. tarda* and *E. ictaluri* isolates. Genome-based phylogenetic analysis revealed that *E. tarda* could be separate into two kinds of genotypes (genotype I, EdwGI and genotype II, EdwGII) based on the sequence similarity. *E. tarda* strains of EdwGI were clustered together with the *E. ictaluri* lineage and showed low sequence conservation to *E. tarda* strains of EdwGII. Multilocus sequence analysis (MLSA) of 48 distinct *Edwardsiella* strains also supports the new taxonomic relationship of the lineages. We identified the type III and VI secretion systems (T3SS and T6SS) as well as iron scavenging related genes that fulfilled the criteria of a key evolutionary factor likely facilitating the virulence evolution and adaptation to a broad range of hosts in EdwGI *E. tarda*. The surface structure-related genes may underlie the adaptive evolution of *E. ictaluri* in the host specification processes. Virulence and competition assays of the null mutants of the representative genes experimentally confirmed their contributive roles in the evolution/niche adaptive processes. We also reconstructed the hypothetical evolutionary pathway to highlight the virulence evolution and niche adaptation mechanisms of *Edwardsiella*. This study may facilitate the development of diagnostics, vaccines, and therapeutics for this under-studied pathogen.

## Introduction

The genus *Edwardsiella*, consisting of three species *Edwardsiella tarda*, *Edwardsiella ictaluri* and *Edwardsiella hoshinae*, was firstly described in 1965 by Ewing *et al* [1] to designate a distinct taxa within the family *Enterobacteriaceae*. *E. hoshinae* is sometimes isolated from animals but its ability to cause disease has not been established and relatively little is known regarding its habitats. *E. ictaluri* is a notorious fish pathogen causing enteric septicemia exclusively in channel catfish (ESC) [1]–[4]. *E. tarda* is the most predominant species as it is a common inhabitant of animals including fish, reptiles, amphibians, chickens, other warm-blooded animals and humans [1], [4], [5], [6]. *E. tarda* is also the etiological agent of edwardsiellosis, characterized by systemic hemorrhagic septicemia, internal abscesses, and skin lesions leading to mass mortality outbreaks in more than 20 species of freshwater and marine fish, causing devastating economic losses in worldwide aquaculture [1], [4]. Moreover, *E. tarda* is also associated with opportunistic infections in humans, most commonly gastroenteritis and wound infections, and sporadic septicemia, meningitis and liver abscess [6], [7], raising a concern that *E. tarda* is becoming a significant zoonotic pathogen that warrants extensive investigation.

The diversity of *E. tarda* isolates in terms of natural niches, geographical dissemination, biochemical and physiological features, and pathogenic properties have been examined using a variety of techniques, including phenotypic analysis, serovar grouping [1], [8], total, extracellular and outer membrane protein profiling [9], plasmids, production of fatty acid methyl esters and antibiotic resistance patterns [10]. PCR-based genetic analysis based on *gyrB* or virulence determinants [11], [12], restriction fragment length polymorphism (RFLP) PCR of 16S rDNA [10], rep-PCR [12]–[15], and PCR ribotyping of 16S-23S spacer genes in rRNA operons were also performed in attempts to group various *E. tarda* isolates [15]. These analytical methods are useful in assessing relatedness of strains but are limited in their resolution between pathogenic strains and environmental isolates, and in their ability to define genetic variances that relate to pathogenicity and phylogenetic significance and offer greater potential for development of practical and reliable diagnostics, vaccines, and therapeutics.

To comprehensively and systematically explore the genetic diversity and virulence evolution of *Edwardsiella* strains, a genome wide profiling is needed. The complete genome sequences of *E. tarda* EIB202 [16], FL6-60 [17], and *E. ictaluri* 93–146 [18] (Table 1) can be used as the reference for comparative genomic analysis. Here we report the sequencing of the genomes of one eel-isolated virulent *E. tarda* strain (080813), one human feces-isolated *E. tarda* type strain (ATCC15947), one freshwater fish-isolated *E. tarda* strain (DT), and one *E. ictaluri* type strain (ATCC33202) using next generation sequencing methods, including Roche 454 and Illumina Solexa (Table 1). We also used the published draft genome sequence of *E. tarda* strain ATCC23685 isolated from human feces for comparative analysis. High-resolution genetic fingerprinting of bacterial isolates will be a valuable tool for distinguishing relapses from new infections, and identifying environmental reservoirs. Furthermore, we performed a genomic survey of gene drifts and positive selection in *Edwardsiella* strains and reconstructed the hypothetical evolutionary pathway to highlight their virulence evolution and niche adaptation mechanisms.

**Table 1:** Strains used in this study and general sequence information of different *Edwardsiella* strains.

Organism	Strain	Classification^a^	Status	Size (Mbp)	ORFS	GC (%)	Origin^b^	Plasmid	Platform	Accession No.
*E. tarda*	EIB202	EdwGI	Complete	3.760	3,563	59.7	Turbot in Yantai, China (2008) [16], [25]	1	454	CP001135
*E. tarda*	FL6-60	EdwGI	Complete	3.684	3,194	59.8	Striped bass in Maryland, U.S.A (1994) [22]	1	454	CP002154
*E. tarda*	ATCC23685	EdwGII	Draft	3.631	3,397	57	Human feces in U.S.A (1959) [21]	NA^c^	454	ADGK00000000
*E. tarda*	080813	EdwGI	Draft	4.296	4,146	58.3	Japanese eel in Fujian, China (2008) [12]	>1	454	AFJH00000000
*E. tarda*	ATCC15947	EdwGII	Draft	3,694	3,351	57.1	Human feces in Kentucky, U.S.A (1959) [19]	NA	Solexa	AFJG00000000
*E. tarda*	DT	EdwGII	Draft	3.759	3,460	57	Oscar fish in Guangzhou, China (2007) [12]	NA	454	AFJJ00000000
*E. ictaluri*	93–146		Complete	3.812	3,783	57.4	Catfish in Louisiana, U.S.A (1993) [24]	0	454	CP001600
*E. ictaluri*	ATCC33202		Draft	3.703	3,617	57.7	Catfish in Georgia, U.S.A (1976) [20]	NA	454	AFJI00000000

a *E. tarda* strains were classified into EdwGI and EdwGII clades according to their sequence similarity and ANI value as detailed in the related text.

b The isolation time of *Edwardsiella* strain are shown within brackets.

c NA indicated that plasmids were not investigated in this study.

## Results

### Selection and phenotypes of *Edwardsiella* strains

With the aim to investigate genome diversity of *Edwardsiella* strains from various natural habitats, we selected four strains isolated from different hosts and different geographic locations of the world and sequenced their genomes with the next generation sequencing methods (Table 1). *E. tarda* 080813 was isolated from diseased Japanese eel in Fujian, China [12]. *E. tarda* DT was isolated from Oscar (*Astronotus ocellatus*) in Guangzhou, China [12]. *E. tarda* ATCC15947 is the type strain of *E. tarda* isolated from human feces in Kentucky, USA [19] and *E. ictaluri* ATCC33202, the type strain of *E*. *ictaluri*, was isolated from diseased channel catfish in Georgia, USA [20]. Three other published *Edwardsiella* genomes were also used in this study, including *E. tarda* ATCC23685 isolated from human feces [21], *E. tarda* FL6-60, a highly virulent strain isolated from a striped bass in Maryland, USA [22], and *E. ictaluri* 93–146, isolated from a commercial catfish pond in Louisiana, USA [23], [24]. The published genome of *E. tarda* strain EIB202, isolated from a diseased turbot (*Scophthalmus maximus*) in Shandong, China, was also included as the reference genome in this study [16], [25].

We assessed the biochemical and growth characteristics of the sequenced *Edwardsiella* strains. While the growth rate of *E. ictaluri* ATCC33202 was markedly lower than that of *E. tarda* strains, there is no significant variation in growth rate among the different strains of *E. tarda* in LB rich medium (data not shown). Based on the API 20E test, *E. tarda* is an easily recognizable species as it produces H_2_S (H2S), ornithine decarboxilase (ODC) and generates indole from tryptophan (IND), while *E. ictaluri* ATCC33202 was negative in these tests as previously described (Table S1) [1].

**Figure 1:**
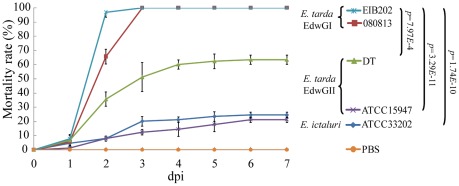
Pathogenic characteristics of *Edwardsiella* strains. Cumulative mortality in zebra fish i.m. challenged with the indicated *Edwardsiella* strains. No mortality was observed after 7 days of observation (data not shown). Error bars showed the standard deviations calculated from three individual experiments.

We used zebrafish as the animal model to investigate virulence characteristics of these strains (Figure 1). Fish injected with 5 µl 1×10^5^ cfu/ml of *E. tarda* 080813 and EIB202 showed 100% cumulative mortality rate at 3 days post infection (dpi), while significant lower mortality rates were obtained for *E. tarda* DT (66%, *p* = 7.97*E*-4), *E. tarda* ATCC15947 (21%, *p* = 3.29*E*-11) and *E. ictaluri* ATCC33202 (24%, *p* = 1.74*E*-10) at 7 dpi. Mortalities due to *Edwardsiella* infection in adult zebra fish began 1 dpi and continued through 5 dpi, after which there were no further deaths. The majority of the mortalities occurred between 1 and 3 dpi. The fish infected by *E. tarda* 080813 and EIB202 exhibited typical symptoms of edwardsiellosis [25], i.e. bleeding in the injection sites, ulceration and necrosis in internal organs and a high bacterial load in the organs as examined by plate count on DHL selection agar. *E. tarda* ATCC15947 and DT as well as *E. ictaluri* ATCC 33202 displayed no or slight clinical signs of infection. Control fish treated with 5 µl PBS showed no mortality or signs of disease over a period of 7 dpi. The group of zebra fish challenged with *E. ictaluri* showed the lowest mortality rate in all these *Edwardsiella* strains, which might be a manifestation of the fact that *E. ictaluri* is almost exclusively associated with ictalurid fish [1].

### General features of sequenced genomes

Sequenced genomes generated 23 to 36-fold coverage (averaged read length ranging from 399 to 428 bp) with 83–117 large contigs (longer than 500 bp) for Roche 454 samples and 80-fold coverage and 159 assembled contigs for the Illumina Solexa sample, respectively (Table 1). The predicted median genome size of sequenced strains is 3,819,423 bp and the average G+C content ranged from 57% to 58.4%, which is similar to that of EIB202 (59.7%). *E. tarda* 080813 contained a higher G+C content (58.38%) than that of *E. tarda* ATCC15947 (57.11%), DT (57.03%) and *E. ictaulut* ATCC33202 (57.56%). RAST subsystem-based annotation identified 3,460 predicted coding sequences (CDSs) in the draft genome of DT, 3,617 in ATCC33202, 3,351 in ATCC15947, and 4,146 in 080813, respectively (Tables 1 and S2). Thus the genome of 080813 stands so far as the largest genome in the sequenced *Edwardsiella* strains. Approx. 20% of CDSs in *Edwardsiella* species were annotated as hypothetical proteins. The overall subsystem category distributions of *E. tarda* strains and *E. ictaluri* strains were similar (Table S2).

### Genomic plasticity of *Edwardsiella* strains

**Figure 2:**
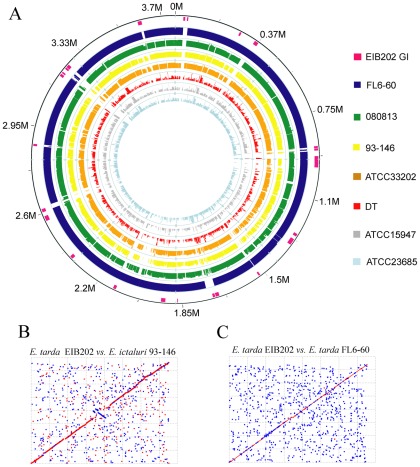
Schematic comparison of the *Edwardsiella* genomes. (A) The outside circle represents *E. tarda* EIB202 GIs (pink). The next 7 circles ranging from outside to inside show the coordinated mapping of 2 complete genomes (*E. tarda* FL6-60 and *E. ictaluri* 93–146) and 5 contig sets of genomes against *E. tarda* EIB202 reference genome sequence. (B and C) Dot plot comparison of MUMmer nucmer output between *E. tarda* EIB202 (x-axis) and *E. ictaluri* 93–146 (y-axis) (B), or between *E. tarda* EIB202 (x-axis) and *E. tarda* FL6-60 (y-axis) (C). Red and blue plot means forward and reverse matches, respectively.

Global pairwise genomic alignment revealed 8 *Edwardsiella* strains could be easily classified into 3 groups as of EIB202-like strains, ATCC15947-like strains, and *E. ictaluri* strains (Figure 2). Nucleotide sequence alignments showed high sequence homology between EIB202 and other EIB202-like *E. tarda* strains (e.g. 080813, FL6-60) (≥94% average sequence identity) (Table S3). EIB202 also showed high sequence similarities with *E. ictaluri* strains (e.g. 93–146, ATCC33202) (92.24%). Sequence alignment revealed 85%∼88% average sequence identity between EIB202-like strains and ATCC15947-like strains (e.g. ATCC15947, ATCC23685, DT) (Figure 2A, Table S3), which was much lower than sequence identity between EIB202 and EIB202-like *E. tarda* strains or between EIB202 and *E. ictaluri* strains. The results showed significant difference among inter-group strains (EIB202-like strains, *E. ictaluri* strains and ATCC15947-like strains) while the intra-group strains did not show significant difference when used *p*<1*E*-3 as threshold (Tables S3). Given the genome-wide diversity between these two types of *E. tarda* strains, in this paper, we termed *E. tarda* EIB202- like strains (EIB202, FL6-60, 080813) as *E. tarda* genotype I (EdwGI) and *E. tarda* ATCC15947-like strains (e.g. ATCC15947, ATCC23685, DT) as *E. tarda* genotype II (EdwGII), respectively.

EIB202 has been established to harbor 24 genomic islands (GIs) [16]. We identified 11 GIs in FL6-60 and 31 GIs in *E. ictaluri* 93–146, respectively (Table S4). Comparison of the GI sequences of EIB202 to that of other EdwGI strains showed that FL6-60 shared most of GI sequences with *E. tarda* EIB202 except GI2, GI12, and GI23, which appear to encode prophage and/or transposase genes (Table S4) [16]. Interestingly, the plasmid pFL6-60 of *E. tarda* FL6-60 contained many (9/63) prophage genes which show high similarity to the prophage and mobile genetic elements in EIB202 chromosome, suggesting that pFL6-60 might be released from the chromosome. *E. tarda* 080813 shared more than half of the GI-like sequences with EIB202, including GI7 that encodes a type III secretion system (T3SS) gene cluster, and GI17 encoding a type VI secretion system (T6SS) gene cluster. Two *E. ictaluri* strains shared most of the GIs between themselves and showed high sequence divergence to *E. tarda* strains in terms of GI content except the GIs for T3SS and T6SS (Table S4).

Thirteen families of IS elements were identified in the sequenced genomes of *Edwardsiella* (Table S4). The most abundant IS elements among these sequenced strains included IS*Kpn2*, IS*102*, IS*200*, IS*Ec30* and partial IS*Saen1*, which are common in the Enterobacteriaceae [26]. There are clearly different types of IS distributed among *E. tarda* and *E. ictaluri* species, while the two *E. ictaluri* strains show the same IS profile. There are 33 complete copies of the IS*1414* element in *E. ictaluri* 93–146 while only one copy of IS*1414* (contain a nonsense mutation TAC to TAA in codon 10 of *tnpA* gene) in *E. tarda* EIB202, which might account for the dormant state of IS*1414* in EIB202. Sequencing results showed that intact IS*1414* is also present in the *E. ictaluri* ATCC33202 draft genome. A partial of IS*1414* is found in *E. tarda* 080813 contigs and the draft sequences of all *E. tarda* EdwGII strains showed no homology to this mobile element, indicating that IS*1414* sequence may exist in ancestral *E. trada* EdwGI and *E. ictaluri* strains.

Taken together, the variance distribution of GI and IS elements in different *E. tarda* strains corresponds to the broad host range properties of *E. tarda* while the conversed GI and IS elements profiles in *E. ictaluri* strains imply that the genomes of different *E. ictaluri* might be kept less modified in relatively fixed hosts.

### Phylogenetic relationships of *Edwardsiella* strains

**Figure 3:**
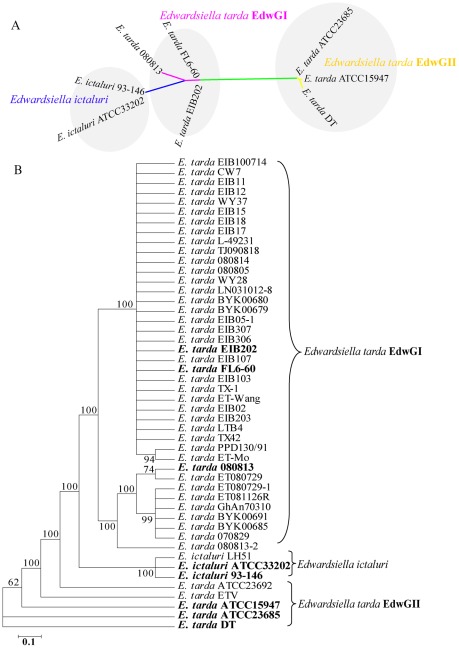
Phylogenetic tree of *Edwardsiella* species. (A) Maximum likelihood phylogeny based on all filtered SNPs across 8 *Edwardsiella* genomes. Branches are colored according to the main phylogeographic lineages of *Edwardsiella* bacteria. (B) NJ tree of 48 *Edwardsiella* strains inferred from concatenated alignments of partial coding sequences of *glyA*, *mdh*, *pgi*, *fusA*, *aspA* and *tpi* genes with 100 bootstrap iterations. Strains investigated in this study are indicated in bold font.

The specific taxonomic position of *Edwardsiella* bacterium in Enterobacteriaceae was previously reached with 44 house-keeping genes [16]. The same method was applied to the sequenced 8 genomes. The result indicated that the 3 EdwGI strains clustered tightly together with the 2 *E. ictaluri* strains, forming a distinct branch and the 3 *E. tarda* EdwGII strains are clustered into another branch (Figure S1). In this study, a genome-wide SNP-based maximum likelihood tree was further constructed using all high confidence SNP sites among the 8 *Edwardsiella* strains. The result demonstrated that *E. tarda* EdwGI and EdwGII strains and *E. ictaluri* strains are clustered into 3 distinct clades and *E. tarda* EdwGI strains are more closely related to *E. ictaluri* strains than to *E. tarda* EdwGII strains (Figure 3A).

Multilocus sequence analysis (MLSA) of 48 collected *Edwardsiella* strains (Table S5) isolated from various hosts at different time also showed that *E. tarda* strains isolated from diseased fish were clustered tightly together with the *E. tarda* EdwGI strains EIB202 and FL6-60, and the majority of *E. tarda* strains from diseased eel were grouped with the *E. tarda* EdwGI strain 080813, forming a larger branch (Figure 3B). The *E. ictaluri* strains could be closely classified as a unique group, while *E. tarda* EdwGII strains are clustered into another distant branch (Figure 3B). All these phylogenetics/phylogenomics relationship of 8 sequenced *Edwardsiall* strains indicating that the genetic relationship of EdwGI *E. tarda* and *E. ictaluri* are closer to each other than that between *E. tarda* strains of EdwGI and EdwGII.

We then estimated the last common ancestor between each pair of genomes based on the pairwise synonymous substitution frequency (Ds) values of ∼1,000 house-keeping genes shared by the 8 genomes. The estimated Ds value was 0.0004 between *E. tarda* EIB202 and FL6-60, 0.0005 between *E ictaluri* ATCC33202 and 93–146, 0.18 between EIB202 and *E ictaluri*, and 0.48 between *E. tarda* EdwGI and EdwGII strains (Table S6). Mirroring the nucleotide-based phylogeny results (Figure 3), *E. tarda* EdwGI and EdwGII strains split from a common ancestor much longer than that for EdwGI *E. tarda* and *E. ictaluri* strains, suggesting a common ancestor might exist for *E. tarda* EdwGI strains and *E. ictaluri* strains.

We further took advantage of the widely used average nucleotide identity (ANI) method introduced by Konstantinidis and Tiedje [27], [28] which transform the ANI values derived from genome sequences into DNA-DNA hybridization (DDH) values traditionally used in species definition. We used the ANI data of the 8 *Edwardsiella* genomes to split the three groups of isolates by using the 94% ANI criterion (equal to 70% DDH value) for assignment of strains to species (Table S6) [28]. The results showed that ANI value of the 3 EdwGI strains were higher than 94% while that between *E. tarda* EdwGI strains and *E. ictaluri* species were ∼92%, demonstrating their close phylogenomic relationships (Table S6). ANI analysis indicated that *E. tarda* EdwGII strains showed more distant relationships to *E. tarda* EdwGI strains (∼82%) and to *E. ictaluri* species (∼82%). Furthermore, the averaged Ds values derived from the housekeeping genes in each pair of the *Edwardsiella* strains showed a tight correspondence to their ANI values (R^2^ = 0.9949) (Table S6, Figure S2), demonstrating that both methods are valid to distinguish different species of *Edwardsiella*.

Taken together, these data support the following phylogenetic inferences. Firstly, 8 *Edwardsella* strains could be grouped into three distinct major lineages. *E. tarda* EdwGI strains form a monophyletic lineage which is the sister clade of *E. ictaluri* strains. Secondly, although some of the *E. tarda* strains isolated from humans (EdwGII) and marine fish (EdwGI) were classified into the same species, these strains might diverge from a common ancestor before the *E. tarda* EdwGI and *E. ictaluri* strains split from each other.

### Distribution of orthologs and specific genes in *Edwardsiella* strains

**Figure 4:**
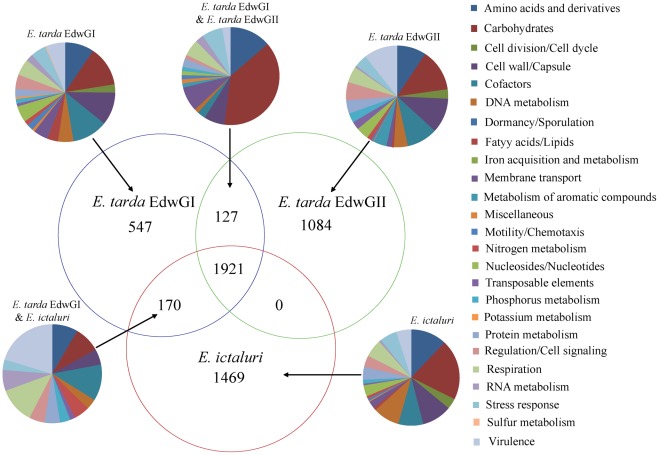
The Venn diagram illustrating the number of genes unique or shared between two *Edwardsiella* lineages. The associated pie charts showed the functional groups assigned for CDSs in relevant sections of the Venn diagram. The strains used for comparison were *E. tarda* EIB202, FL6-60, and 080813 in EdwGI lineage, DT, ATCC15947, and ATCC23685 in EdwGII lineage, and *E. ictaluri* ATCC33202 and 93–146.

Comparison of the genome sequences revealed that 1,921 distinct genes were shared by the 8 *Edwardsiella* strains (Figure 4, Table S7). Another 844 orthologs were identified in 3 *E. tarda* EdwGI strains. Other 1,211 orthologs were shared by 2 *E. ictaluri* strains and 1,639 homologs in 3 *E. tarda* EdwGII strains, respectively (Figure 4, Table S7). Hence, the core gene set (1,921) may represent about 36.1% of all distinct genes identified in the 8 genomes (Figure 4). The following genes were highlighted to pertain to different clusters of *Edwardsiella* strains.

*E. tarda* contain 127 genes whose sequences are absent in the genome of *E. ictaluri* strains (Figure 4, Table S7). These genes include *tnaA* and *tnaB* for indole production which is one of the differential phenotypes for *E. tarda* and *E. ictaluri* (Table S1) [1]. The genes also include *pvsA*, *pvsD*, *pvsE*, and *pvuA*, encoding siderophore vibrioferrin biosynthesis and transport related proteins that play essential roles in a unique iron acquisition system originally identified in marine bacteria *Vibrio parahaemolyticus*, *V. alginolyticus*, and *V. splendidus* [29], [30], presumably endowing *E. tarda* species survival and propagation advantages in the marine environment and other iron-restricted environments. Several genes encoding two component system (TCS) are specific to *E. tarda*, including *yehT*/*yehU* involved in deoxycholate and crystal violet resistance [31], a potential *pleC*/*pleD* system involved in intracellular infection [32], and the *lytR*/*lytS* system implicated to be involved in bacterial stress responses [33]. These lineage-specific genes might underlie the differentiation of the host-adaptation processes of *E. tarda* and *E. ictaluri*.

*E. tarda* EdwGI strains and *E. ictaluri* strains shared a wide range of genes involved in host interaction and virulence, including T3SS and T6SS (Table S8). Previous reports showed that *E. tarda* T3SS and T6SS gene clusters consist of 32 and 16 genes, respectively [16], [34], [35]. The T3SS and T6SS genes in EdwGI strains and *E. ictaluri* strains are highly homologous to the previously described counterparts in *E. tarda* strain PPD130/91 [18], [36], [37]. Examination of T3SS and T6SS homologs in EdwGI strains and *E. ictaluri* strains showed the same genetic organization and shared 78%–100% amino acid sequence identity (Figure S3, Table S8). T6SS secreted protein EvpP [18], [36], [37] displayed the highest genetic diversity (78%–91% amino acid sequence identity) among the EdwGI strains and *E. ictaluri* strains (Table S8). Notably, *E. tarda* EdwGII strains lost most of the T3SS and T6SS orthologs (Figures S3, Table S8), indicated that some important virulence factors were missing in *E. tarda* EdwGII strains ATCC15947, DT, and ATCC23685. The NJ-based tree of 6 *Edwardsiella* isolates (3 EdwGI strains, 2 *E. icatluri* strains and *E. tarda* PPD130/91) (Figure S3C) and the MLSA result (Figure 3B) indicated that PPD130/91 could be classified into EdwGI. Encoded in the T3SS gene locus, EsrA/EsrB was established to be responsible for regulation of the T3SS and T6SS in *E. tarda* [34], [36], [37]. Sequence analysis of EsrA/EsrB genes of *Edwardsiella* strains showed the same phylogeny topology to that of the phylogenetic tree inferred by house-keeping genes (Figures 3 and S3D).

**Figure 5:**
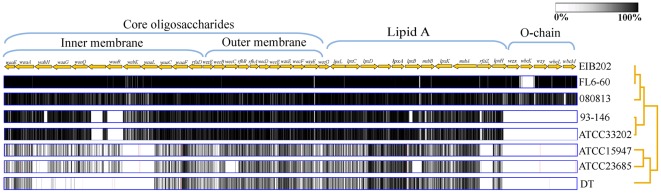
LPS related genes of *Edwardsiella* strains. Mauve progressive alignment of the concatenated coding sequences of 8 sequenced *Edwardsiella* strains using *E. tarda* EIB202 as reference. Arrows indicate the gene coding orientation in EIB202 genome. The dendrogram is derived from NJ analysis of concatenated amino acid sequences of LPS biosynthesis genes with 1,000 bootstrap iterations. Gradient bar indicated the sequence similarity of LPS coding sequences of *Edwardsiella* strains to those of EIB202.

Previous serotyping schemes have recognized more than 61 O groups and 45 H antigens in *E. tarda* [38] while *E. ictaluri* isolates from enteric septicemia of catfish (ESC) outbreaks are all of the same serotype [39]. The genetic distance of the predicted LPS genes of the *Edwardsiella* strains (Figure 5) was largely consistent with their phylogenetic tree (Figure 3). *E. tarda* EdwGI strains and *E. ictaluri* strains share a majority of LPS genes except *waaR*, encoding a core glycosyl transferase and some genes involved in O-antigen synthesis (*wzx*, *wbcK*, *wzy*, *wbcL*, and *wbcM*) (Figure 5, Table S9) [40], implying a genetic basis for LPS or O-serotype variations between the host-specific and broad host-range strains. Moreover, high sequence diversity was observed in the genes for inner core oligosaccharides and O-chain between *E. tarda* EdwGI and EdwGII strains (Figure 5). These variable regions include the *waaL* gene required for production of high molecular weight O-antigen side chains in *E. tarda* [41] (Table S9). Interestingly, the O-antigen gene cluster of *E. tarda* EdwGII strains (ATCC15947 and ATCC23685) isolated from human feces showed more sequence similarities to *E. coli* than to other *Edwardsiella* strains, suggesting a putative human gut adaptation process of these bacteria (Table S9).

### Polymorphisms and positive selection in *Edwardsiella* core genomes

**Figure 6:**
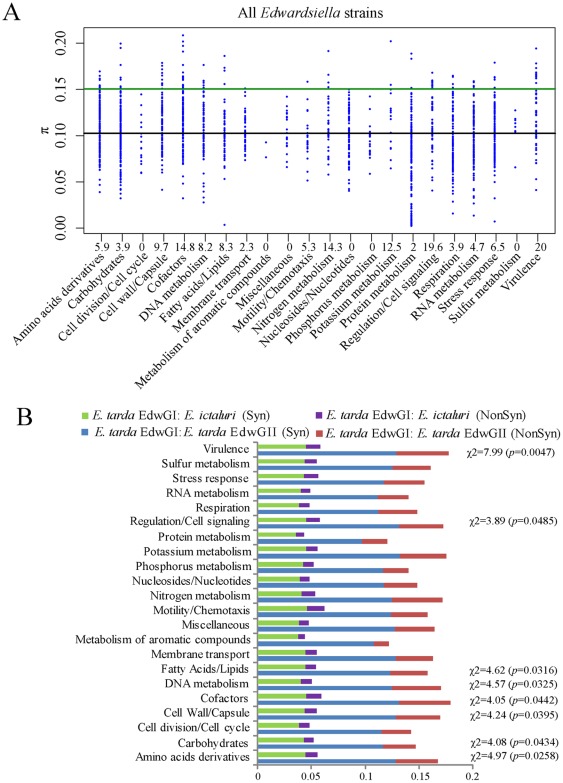
Genome-wide nucleotide variations among the orthologs of sequenced *Edwardsiella* strains. (A) Nucleotide diversity (π) for 8 *Edwardsiella* strains. The black line represents the average π value of all orthologs. Green line indicates π values above 1.5σ (standard deviation) from the average π values of all orthologs, respectively. The percent of genes with π values large than 1.5σ from the average π value in each function category are shown under x axis. (B) Analysis of the ratio of nonsynonymous (NonSyn) to synonymous (Syn) SNP rates according to the RAST-annotated categories. The set of genes which contain significant high ratios (*p*<0.05) of nonsynonymous (NonSyn) SNPs than synonymous (Syn) SNPs between *E. tarda* EdwGI and *E. tarda* EdwGII strains were as indicated.

To understand the level and nature of nucleotide variation among all 8 sequenced *Edwardsiella* genomes, nucleotide diversity (π) of 1921 aligned orthologous sequences were calculated (Figure 6A, Table S10) [42], [43]. Although these 8 genomes were clustered into 3 distinct phylogenetic clades, most of the orthologs involved in cell cycle, membrane transport and nucleotides/RNA metabolisms showed a high degree of conservation and less than 5% orthologs displayed significantly greater (>1.5 standard deviation (σ)) π values than the mean π value among these lineages (Figure 6A). In contrast, high percentage of homologous genes related to the RAST-defined functions in cell wall and capsule (9.7%), cofactors (14.8%), nitrogen metabolism (14.3%), regulation and cell signaling (19.6%), and virulence (20%) exhibited significantly high nucleotide diversity (>1.5σ above the mean π value) among these *Edwardsiella* genomes (Figure 6A). Nucleotide diversity calculation was also performed with EdwGI/EdwGII (Figure S4A) and with EdwGI/*E. ictaluri* (Figure S4B) orthologs. The results indicated that EdwGI and EdwGII genomes share significantly high diversity (>1.5σ) in cell wall and capsule (10.6%), cofactors (12.9%), regulation and cell signaling (14.8%), and virulence (21.1%) (Figure S4A) while that between EdwGI strains and *E. ictaluri* mainly focus on membrane transport (11.4%), motility and chemotaxis (17.4%), nitrogen metabolism (14.3%), regulation and cell signaling (11.1%), and virulence (12.3%) (Figure S4B).

We further compared the proportion of nonsynonymous (NonSyn) changes in different functional groups of gene sets between EdwGI/*E. ictaluri* and EdwGI/EdwGII strains [42], [43]. When use EIB202 as reference, we found that the ratios of NonSyn changes between EdwGI and EdwGII strains were significantly different in some function categories (Figure 6B), including cell wall and capsule (*p* = 0.0395), regulation and cell signaling (*p* = 0.0485), and virulence (*p* = 0.0047), which were consistent with the detected categories with high nucleotide diversity (Figures 6A and S4A).

A molecular adaptation analyses was performed with 1,921 *Edwardsiella* orthologs to detect gene displaying features of differential selective pressure (positive selection) using two different positive selection models (Branch and Site models) in PAML package [44]. 136 and 129 genes were shown to be under positive selection when used *E. tarda* EdwGI and *E. ictaluri* strains as foreground branches, respectively (*p*<0.05, likelihood ratio test, LRT) (Tables 2 and S11). In particular, thirteen iron uptake and utilization related genes, which were classified as virulence related genes according to the RAST function catalogs, were significantly enriched in gene set (*p* = 2.18*E*-13, FDR q value = 5.67*E*-12) in *E. tarda* EdwGI strains. These genes included *hemX*, *hemC*, *hemD*, *hemM*, *hemN*, *hemS*, *hemT* and ETAE_1794, a ChuX-like heme iron utilization protein [45], ETAE_2768-2770, an iron transport related ABC transporter system, as well as *fur* and *basS*, two genes involved in iron uptake [46] (Table 2). Another group of genes subjected to high selection pressure in EdwGI strains (Table 2) are genes required for responses to environmental stresses including *phoR* (response to phosphate starvation) [47], *gor* (oxidative stress response) [48], *envZ* (osmolarity stress regulation) [49], and *pspF* (involved in responses to ethanol, osmotic shock, and heat shock) [50]. The widespread presence of positive selection sites in iron acquisition-related genes and signal response-related genes indicated their essential roles for the *E. tarda* EdwGI strains to inhabit different environment niches. Similarly, a significantly large number of surface structure (cell wall and capsule/motility and chemotaxis) related genes under positive selection were enriched in *E. ictaluri* (n = 14, *p* = 9.91*E*-8, FDR q value = 8.54*E*-6), including flagellar biosynthesis genes *flhB*, *flhA*, *motA*, *fliG*, and *fliR* and the LPS assembly related gene *imp*, membrane associated proteins such as Tol-Pal system-related genes *tolB/tolC* [51], the penicillin-binding protein encoded by *mrcB*, and the outer membrane protein gene *ompW* [52]. The selection of these surface related structures might have specifically contributed to the adaptation processes of the bacterium to the channel catfish host.

**Table 2:** Representative genes with high diversity or under positive selection.

CDS	Gene	RAST catalog	Pi^a^	*E. tarda* EdwGI		*E. ictaluri*	Annotation
				Chi^b^	LRT^c^	LRT	
ETAE_0116	*hemX*	Virulence		*	*		Uroporphyrinogen III C-methyltransferase
ETAE_0117	*hemD*	Virulence	0.153	*	*		Uroporphyrinogen-III synthase
ETAE_0118	*hemC*	Virulence		*	**		Porphobilinogen deaminase
ETAE_0271	*hemN*	Virulence		*	**		Fe-S oxidoreductases
ETAE_1404	*hemM*	Virulence			**		Outer membrane lipoprotein
ETAE_1798	*hemS*	Virulence		*			Hemin transport protein
ETAE_1799	*hmuT*	Virulence	0.168	*	**		Hemin-binding periplasmic protein
ETAE_1794		Virulence	0.157	*	*		Heme iron utilization protein
ETAE_2610	*fur*	Virulence		*			Ferric uptake regulator
ETAE_2768		Virulence		*			ABC transporter, substrate binding protein
ETAE_2769		Virulence		*			ABC transporter, permease protein
ETAE_2770		Virulence		*			ABC transporter, ATP-binding protein
ETAE_0393	*basS*	Regulation and cell signaling	0.154		*		Sensor protein BasS/PmrB
ETAE_1081	*phoR*	Other			*		Phosphate regulon sensor protein
ETAE_3367	*gor*	Stress response			*		Glutathione-disulfide reductase
ETAE_3278	*envZ*	Other			*		Osmolarity sensor protein
ETAE_1242	*pepN*	Stress response			*		Aminopeptidase N
ETAE_1868	*pspF*	Stress response			**		Phage shock protein F
ETAE_2912		Regulation and cell signaling	0.159		**		Transcriptional regulator, LysR family
ETAE_2767	*emrB*	Other			**		Multidrug resistance protein B
ETAE_1219	*flhB*	Motility and chemotaxis				**	Flagellar biosynthetic protein
ETAE_1220	*flhA*	Motility and chemotaxis				*	Flagellar biosynthesis protein
ETAE_1338	*motA*	Motility and chemotaxis				*	Flagellar motor protein
ETAE_2143	*fliG*	Motility and chemotaxis				*	Flagellar motor switch protein
ETAE_2154	*fliR*	Motility and chemotaxis				*	Flagellar biosynthesis pathway component
ETAE_0191	*tolC*	Virulence				**	Outer membrane protein
ETAE_2573	*tolB*	Virulence				*	Translocation protein
ETAE_1528	*ompW*	Other				*	Outer membrane protein W
ETAE_0263	*mltC*	Cell wall and capsule				*	Murein transglycosylase C
ETAE_0382	*yjfG*	Cell wall and capsule				**	UDP-N-acetylmuramate
ETAE_0603	*imp*	Cell wall and capsule				*	LPS-assembly protein
ETAE_0695	*mrcB*	Cell wall and capsule				*	Penicillin-binding protein
ETAE_1032		Cell wall and capsule				**	Fimbrial usher protein
ETAE_1126	*amiA*	Cell wall and capsule				**	N-Acetylmuramoyl-L-alanine amidase

a Nucleotide diversity value (π) of orthologs of sequenced *Edwardsiella* strains [43]; Representative genes which differed by above 1.5 σ from the average π value of all orthogolous were listed.

b χ^2^ test of nonsynonymous (NonSyn) changes of *E. tarda* EdwGI strains/*E. ictaluri* and *E. tarda* EdwGI/EdwGII lineages.

c LRT test of branch and site models in PAML package [44].

* *p*<0.05;

** *p*<0.01.

The detailed *p* value and other information were shown in Tables S10 and S11.

### Strain specific and positively selected genes contribute to virulence and adaptation

**Figure 7:**
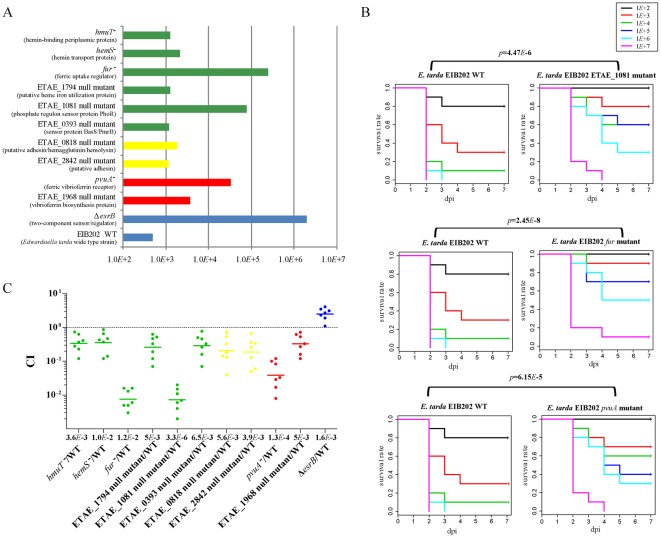
Contributive roles of representative diversified or positive selection genes in the virulence and colonization in zebra fish. (A) LD_50_ values of the wild-type EIB202 (WT) and the null mutants of the indicated genes. LD_50_ is calculated by the method described elsewhere [36]. (B) Virulence comparison of parental *E. tarda* EIB202 with the mutants with gene disruption in ETAE_1081, *fur*, and *pvuA*, respectively. Graphs show survival curves of zebra fish following injected with varying dosages of *E. tarda* strains. All mutant strains are significantly attenuated compared to parental EIB202 strain (*p*<0.01, Mantel-Haenszel Chi-squared test). (C) Competitive indexes of the indicated strains against WT in zebra fish at 24 h after inoculation. WT was differentiated from the mutant strain based upon GFP label or Km resistance on DHL agar plates as detailed in the Materials and Methods [37]. The strain Δ*esrB* with significantly attenuated virulence while transitorily enhanced CI was included in the experiments as a control [36], [37]. The *p* value of the decreased growth competition of the mutants against WT are shown under x axis (*p*<0.01, one sample *t-*test).

We were intrigued by the possibility that the strain specific genes and the positively selected genes might contribute to the colonization and virulence towards the hosts. Previous findings have demonstrated that the T3SS and T6SS are essential for the virulence of *E. tarda* and *E. ictaluri* [34], [35], [37], [53]. To evaluate if other strain-specific genes and positively selected genes might be involved in the host virulence and colonization, we selected 10 representative genes (2 *E. tarda* specific genes, 2 *E. tarda* EdwGI strain-specific genes, 6 positively selected genes in EdwGI strains) (Figure 7, Tables 2 and S7) and generated isogenic *E. tarda* mutant strains to test the LD_50_ values and competition index in zebra fish model. Compared to the parental *E. tarda* EIB202, all the mutants exhibited 2.3 to 504 fold attenuation in virulence (Figure 7A). The cumulative mortality of the mutant strains with the gene disruption in ETAE_1081, *fur*, and *pvuA* showed significantly decreased virulence when compared with that of parental *E. tarda* EIB202 (*p*<0.01) (Figure 7B), indicating that these genes play critical roles in the invasion process in fish. All the mutant strains displayed significantly decreased growth competition against the wild-type strain (Figure 7C). The *ΔesrB* mutant strain, which was found to inhibit the expression of T3SS and T6SS while activate hemolysin EthA production, displayed 4000-fold virulence attenuation and transitorily slightly enhanced competition index [37], [54] (Figure 7C). These results indicated that a subset, if not all, of the diversified and positively selected genes may influence virulence evolution and adaptation processes in *Edwardsiella*.

## Discussion

In this study, we presented for the first time a genome-wide comparative analysis of various *Edwardsiella* isolates pertaining to *E. tarda* EdwGI and EdwGII and *E. ictaluri* lineages as evaluated relative to their genome sequences. The genomic comparison and positive selection model analysis between *E. tarda* EdwGI and EdwGII strains, and *E. ictaluri* strains help to explain the differences in host range and pathogenesis among these three groups of closely related organisms and show potential key gene contents facilitating adaptation in different lineage of *Edwardsiella* strains. The low level of virulence in the *E. tarda* EdwGII lineage could be explained by the missing of some important virulence associated gene clusters such as T3SS and T6SS (Figures S3A and S3B, Table S8), as observed in the previous work where low virulence phenotypes were associated with deletions or other mutations in T3SS and/or T6SS [34], [35], [36], [55]. While the high virulence of *E. tarda* EdwGI strains in zebrafish could be due to the pool of genes involved in host-pathogen interactions, stress responses and adaptation to various hosts (Table S7). Moreover, the function comparison analysis of the genes in *E. tarda* EdwGI and EdwGII strains revealed a high diversity of cell wall/capsule-, regulation/cell signaling- and virulence-related genes (Figure S4), suggesting that this may constitute a genetic basis for the different niche adaptation characteristics and virulence mechanisms of these two *E. tarda* lineages. Specifically, many iron scavenging related genes were detected among the virulence genes under positive selection, showing strong signs of adaptive evolution in the *E. tarda* EdwGI lineages (Table 2). Mutational analysis of these genes really demonstrated their essential roles in virulence and colonization (Figure 7). Taken together, T3SS and T6SS as well as iron scavenging related genes thus fulfilled the criteria of a key evolutionary factor likely facilitating the virulence evolution and adaptation to a broad range of hosts in the *E. tarda* EdwGI strains.

Compared to the *E. tarda* strains with a broad host-range, the *E. ictaluri* strains share the freshwater ictalurid fish as their monomorphic host [1]–[4]. Correspondingly, the gene contents in the *E. ictaluri* strains are highly conserved (Figures 2 and 4, Tables S3 and S7). The loss of the biosynthetic and uptake gene clusters for the siderophore vibrioferrin, which is specific to the most abundant marine bacteria *V. alginolyticus*, *V. parahaemolyticus* and *V. splendidus* [29], [30], may be an important factor restricting the habitats of *E. ictaluri* species to freshwater fish. Moreover, evolution selection analysis showed that the genes for surface structures including flagellar biosynthesis and cell wall and capsule are under an adaptive evolution process, which might constitute one of the adaptive traits in *E. ictaluri* (Table 2).

**Figure 8:**
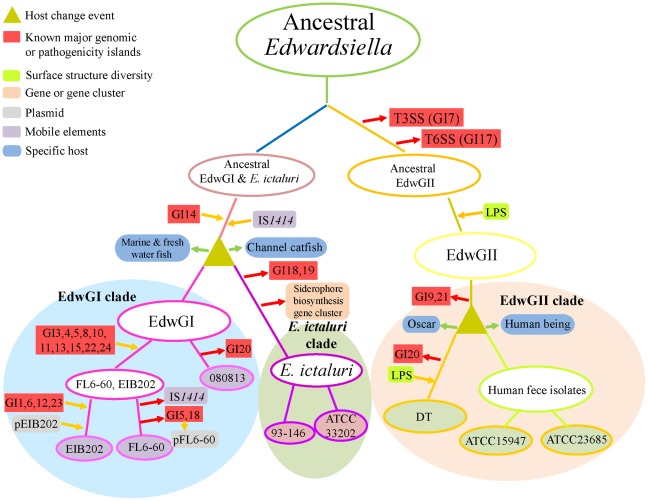
Proposed hypothetical evolutionary pathway of *Edwardsiella* species. Probable insertions, deletions of GIs and gene clusters found in 8 *Edwardsiella* strains are indicated by yellow and red arrows, respectively. Host change events of different strains are indicated by green arrows. Hypothetical ancestral strains are indicated by open circles.

Exploration of the genome content of the strains will definitely provide clues enabling us to track and reconstitute the evolutionary events in *Edwardsiella*. We proposed hypothetical evolutionary scenarios for the *Edwardsiella* strains (Figure 8). Over long periods of time, the large scale changes and microevolution events, including genomic island acquisition and deletion, lateral gene transferring, and mutation accumulation in the genomes, have driven the dynamic modifications of the genome content. On the other hand, various environmental factors such as growth temperatures, osmolarity, and iron limitation etc. have served to select and shape the gene contents in the evolution and adaptation processes of *Edwardsiella* populations. Unknown changes in hosts might have led ancestral *Edwardsiella* clones to diverge into two major subpopulations, which subsequently developed into two distinct clades (*E. tarda* EdwGI lineage and *E. ictaluri*) and one nonpathogenic or environmental clade (*E. tarda* EdwGII lineage) (Figures 3 and 8).

In conclusion, the widely used next generation sequencing methods make it is possible to rapidly identify new genes, gene loss, lineage-specific sequences, darwinian selection and even bacteria adaptation evolution processes underlying the different virulence or niche adaptation features of pathogens, to reconstitute the genetic series of events associated with pathogen evolution, and to trace a specific kind of etiological agent in epidemic outbreaks. Evolutionary parallelism of *Edwardsiella* lineages provides a model to study evolutionary diversity processes linked to the virulence divergence and niche adaptation of pathogenic microorganisms. This approach may facilitate the development of reliable and useful diagnostics, vaccines, and therapeutics for less studied pathogens.

## Experimental Procedures

### Ethics statement

The animal work presented here was approved by the Animal Care Committee, East China University of Science and Technology (approval ID: 2006(272)).

### Bacterial strains

All *E. tarda* strains were grown overnight at 28°C in Luria-Bertani (LB) medium or desoxycholate hydrogen sulfide lactose (DHL) plates. *E. ictaluri* ATCC33202 was grown for 48 h at 25°C in Brain Heart Infusion (BHI) medium with shaking. For API 20E index experiments (bioMérieux, France, Marcy l'Etoile, France), *Edwardsiella* colonies were emulsified into 5 ml of sterile 0.9% NaCl and inoculated into strips according to the instructions provided by the manufacturer.

### Construction of null mutant strains

Insertional null mutants were generated as previously described [37] in *E. tarda* EIB202. Internal fragments of the target genes were obtained by PCR using the primers (Table S12) and treated with BglII/SphI restriction enzymes and cloned into the corresponding restrict sites of pNQ705-1 [56] carrying a kanamycin (Km) and chloramphenicol (Cm) resistance genes. The derivative plasmids were conjugated into EIB202 from *Escherichia coli* SM10 *λpir*. The insertion of the plasmid into each gene of *E. tarda* EIB202 was confirmed by PCR analysis with specific primer pairs (Table S12). Stability of the insertion mutation was tested by growth for 30 generations in the absence of Km as previously described [56].

### Pathogenicity test

Healthy zebra fish weighing ∼0.25 g were acclimatized for 2 weeks in a laboratory breeding system. Aquaria were supplied with flow-through dechlorinated and continuously aerated water at a rate of ∼0.5 L/min. Water temperature was maintained by a central heater at 22±2°C. The fish were reared with a photoperiod of 12∶12 h (light/dark). Pathogenicity was defined by the mortality rate of infected zebra fish. Three paralleled groups of 30 fish were injected intramuscularly (i.m.) with 5 µl bacterial suspension of 1×10^5^ cfu/ml after being sedated in 100 mg/L tricaine methanesulfonate (MS-222, Sigma). Three paralleled control groups of 30 zebra fish were i.m. injected with 5 µl PBS with the same MS-222 treatment. All injected zebra fish were observed for a period of 14 days. The fish deaths caused by *Edwardsiella* strains were confirmed by isolation and re-injection of the strains into zebra fish. The LD_50_ values of all strains were determined in zebra fish as previously described [36]. Competitive index (CI) of the wild-type *E. tarda* EIB202 (WT) and Δ*esrB* strain was performed as previously described by using one-half of the EIB202G harboring a GFP reporter and one-half of the Δ*esrB* strain inoculum (1×10^5^ CFU/ml of each strain) [36], [37]. Seven zebra fish used were sacrificed at 24 h post infection, and were grinded and plated on DHL agar to determine the bacterial loads. WT strain was differentiated from the mutant strain based upon GFP label [37]. The ratios of Δ*esrB* strain counts to WT were used to determine the competitive index. The CI values of other mutants against WT were determined in the same way except being plated on the DHL plates containing Km or DHL plates only for discrimination of the mutants or WT.

### High density sequencing and assembly of genomes

Bacterial genomes were sequenced using the next generation sequencing platforms, Roche 454 (GS FLX Titanium) system and Illumina Solexa Hiseq 2000 system. Large contigs were assembled by using the Newbler *de novo* assembler package for 454 samples. For each Solexa sample, pair-end reads were assembled using Velvet with various values of “hash length” and “cutoff” set by a local Perl script [57]. The quality recalculation process of contigs was performed with Perl script implemented in Consed package [58].

### Genome annotation and comparative genomics

Newly sequenced draft genome sequences were first annotated by using automated prokaryotic annotation pipeline server RAST [59] and then check manually by search against nr protein database using Blastp (E-value cutoff as 1*E*-10 and 60% minimum amino acid sequence identity). We also evaluated the annotation accuracy by comparison the RAST gene calling result of initial *E. tarda* EIB202 454 contigs and simulated Solexa reads of EIB202 genome sequence (assembled by Velvet [57]) with published EIB202 CDSs (CP001135), respectively. More than 92% CDSs were shared in all three kinds of sequences. Most of the CDSs (∼7%) lost in RAST annotation result were putative transposon and prophage related genes, which were excluded in this study. Orthologs of 8 strains were determined by using the best bidirectional Blastp search against EIB202 and query sequences with E-value less than 1*E*-10 and identity more than 60%, matching at least 80% of the length of both query and subject sequences. Genome islands (GIs) and IS elements were predicted by Island Viewer [60] and IS finder [61], respectively. For draft sequences, we identified mobile elements by using IS finder and the absent of these elements in different strains were verified by using PCR method. NUCmer was used for alignment of multiple complete and draft genome sequences with *E. tarda* EIB202 as the reference genome. Genome comparative circular maps were constructed by using GenomeViz package using the NUCmer-coords result files [62], [63].

### SNP calling

MUMmer [62] and NCBI Blastn were used to align large query contigs to the finished EIB202 reference sequence and to generate primary SNP calls. Pseudogene, repetitive sequences, including variable number tandem repeats, single-base insertions or deletions and prophage-related and insertion sequences were excluded from this analysis. SNPs in homopolymeric sequences or Phrap quality low than 40 were also automatically removed by local Perl scripts.

### Phylogeny of *Edwardsiella* species

All filtered SNPs (coding and noncoding SNPs) of 8 *Edwardsiella* strains were used to infer the phylogenetic relationships of *Edwardsiella* strains using maximum likelihood method with 100 bootstrap pseudoreplicates for clade supported by PhyML package. MLSA of 48 *Edwardsialla* isolates (Table S5) was conducted using the concatenated alignment sequences of 6 house-keeping genes (*glyA*, *mdh*, *pgi*, *fusA*, *aspA* and *tpi*) by MEGA5 program with 100 bootstrap iterations for clade support [64]. The ANI values between the query genome and the reference genome were calculated by the Perl script provided by Konstantinos and Tiedje [27].

### SNP analysis

SNAP package was used to obtain the observed synonymous (Syn) substitutions and non-synonymous (NonSyn) substitutions [65]. Gene-by-gene genetic diversity (π) among all *Edwardsiella* strains according to the RAST subsystem category was calculated using Variscan [66]. Omega value (ω = dN/dS, where dN and dS are the nonsynonymous and synonymous substitution rates, respectively) was used to analyze the selective pressures acting on *Edwardsiella* orthologous genes. We first fitted different evolutionary branch models to analyze the ω value among the *E. tarda* EdwGI, *E. tarda* EdwGII and *E. ictaluri* lineages in the phylogenetic tree generated by PhyML (Figure 3A) using the codoml module implemented by PAML (4.4c) program [44]. We also used the site-model of codeml module in the PAML package to detect positive selection sites for aligned genes by calculating likelihood ratio test (LRT) value of model M2a (positive selection) *vs.* model M1a (nearly neutral) and M8 (beta & ω) *vs.* M7 (beta), respectively [44].

### Statistical analysis

Chi-squared (χ^2^) test and Mantel-Haenszel Chi-squared test were used for comparisons of the mortalities of zebra fish infected with *E. tarda* EIB202, sequenced *Edwardsiella* strains, and other *E. tarda* EIB202 mutant strains. Difference of sequence identity was analyzed by one-way ANOVA analysis and Tukey's HSD test. Chi-squared (χ^2^) test was also used to determine whether the proportion of NonSyn changes in various groups of genes showed significant differences in different *Edwardsiella* lineages. An independent one-sample *t*-test was used to determine whether CI values of the mutants against the wild-type strain were significantly different to the log transformation of CI value 0, the expected value implying that there would be no difference between wide-type strain and the mutant strain. Function enrichment was calculated using the hypergeometric distribution at a significance cutoff of ∼5% false discovery rate (FDR). All statistical analysis was performed using R program.

### Data Availability

The nucleotide sequence of the draft sequences were submitted to the GenBank database under accession numbers AFJH00000000 (*E. tarda* 080813), AFJG00000000 (*E. tarda* ATCC15947), AFJJ00000000 (*E. tarda* DT) and AFJI00000000 (*E. ictaluri* ATCC33202), respectively. Sequences used for the multilocus sequence analysis were available under the accession numbers JN709499-JN709721.

## Supporting Information

Figure S1 **Phylogenetic tree of** ***Edwardsiella*** **species.** Phylogenies of *Edwardsiella* species inferred from concatenated alignments of the protein sequences encoded by 44 house-keeping genes (*adk*, *aroC*, *dnaA*, *dnaK*, *frr*, *fusA*, *gapA*, *gyrA*, *gryB*, *infC*, *nusA*, *pgk*, *phoB*, *phoR*, *pyrG*, *recC*, *rplA*, *rplB*, *rplC*, *rplD*, *rplE*, *rplF*, *rplK*, *rplL*, *rplM*, *rplN*, *rplP*, *rplS*, *rplT*, *rpmA*, *rpoA*, *rpoB*, *rpoC*, *rpoE*, *rpsB*, *rpsC*, *rpsE*, *rpsI*, *rpsJ*, *rpsK*, *rpsM*, *rpsS*, *smpB*, *and tsf*) by PhyML program with 100 bootstrap iterations for clade support. *Bacillus cereus* ATCC14579 was used as the outgroup strain.
(TIF)

Figure S2 **Relationships between ANI and synonymous nucleotide substitutions.** Each blue square represents the ANI of all genome sequence between two strains (x axes) plotted against (y axes) the average rate of synonymous nucleotide substitutions of housekeeping genes.
(TIF)

Figure S3 ***Edwardsiella*** **virulence gene clusters of secreted proteins.** T6SS (A) and T3SS (B) gene clusters of sequenced *E. tarda* EdwGI and *E. ictaluri* strains. All genes with high similarity are indicated in the same color and the gene names are shown below according to the color scheme. (C) NJ-tree of 6 *Edwardsialla* isolates (3 EdwGI strains, 2 *E. icatluri* strains and *E. tarda* PPD130/91) inferred from concatenated T6SS and T3SS aligned sequences. (D) NJ tree of 8 *Edwardsiella* species inferred from concatenated alignments of the coding sequences of *esrA* and *esrB* genes with 1000 bootstrap iterations.
(TIF)

Figure S4 **Nucleotide diversity (π) of orthoglous of** ***Edwardsiella*** **.** (A) Nucleotide diversity (π) for *E. tarda* EdwGI and EdwGII. (B) Nucleotide diversity (π) for *E. tarda* EdwGI and *E. ictaluri* strains. The blank line represents the average π value of all orthologs. Green line indicates π values above 1.5σ (standard deviation) from the average π values of all orthologs, respectively. The percent of genes with π values large than 1.5σ from the average π value in each function category are shown under x axis.
(TIF)

Table S1 **API-20E test of** ***Edwardsiella*** **strains.**
(DOC)

Table S2 **RAST annotation of the genomes of the** ***Edwardsiella*** **strains.**
(XLS)

Table S3 **Sequence identity of 8** ***Edwardsiella*** **strains.**
(DOC)

Table S4 **Predicted GI related sequences and IS distribution in the genomes of the** ***Edwardsiella*** **strains.**
(XLS)

Table S5 **Multilocus sequence analysis of 48 strains.**
(XLS)

Table S6 **ANI value and synonymous substitution frequency of the strains studied.**
(DOC)

Table S7 **Ortholog distribution in the genomes of the** ***Edwardsiella*** **strains.**
(XLS)

Table S8 **Amino acid sequence identity of the T3SS and T6SS between the** ***Edwardsiella*** **strains.**
(XLS)

Table S9 **LPS biosynthesis related genes in the** ***Edwardsiella*** **strains.**
(XLS)

Table S10 **Nucleotide variation value pi of the** ***Edwardsiella*** **strains.**
(XLS)

Table S11 **positive selected genes.**
(XLS)

Table S12 **primer list.**
(XLS)
