# Supplementary figures and images for: Edwardsiella Comparative Phylogenomics Reveal the New Intra/Inter-Species Taxonomic Relationships, Virulence Evolution and Niche Adaptation Mechanisms
Source: PLoS One. 2012 May 10;7(5):e36987. doi: 10.1371/journal.pone.0036987 (PMC3349661; doi:10.1371/journal.pone.0036987)

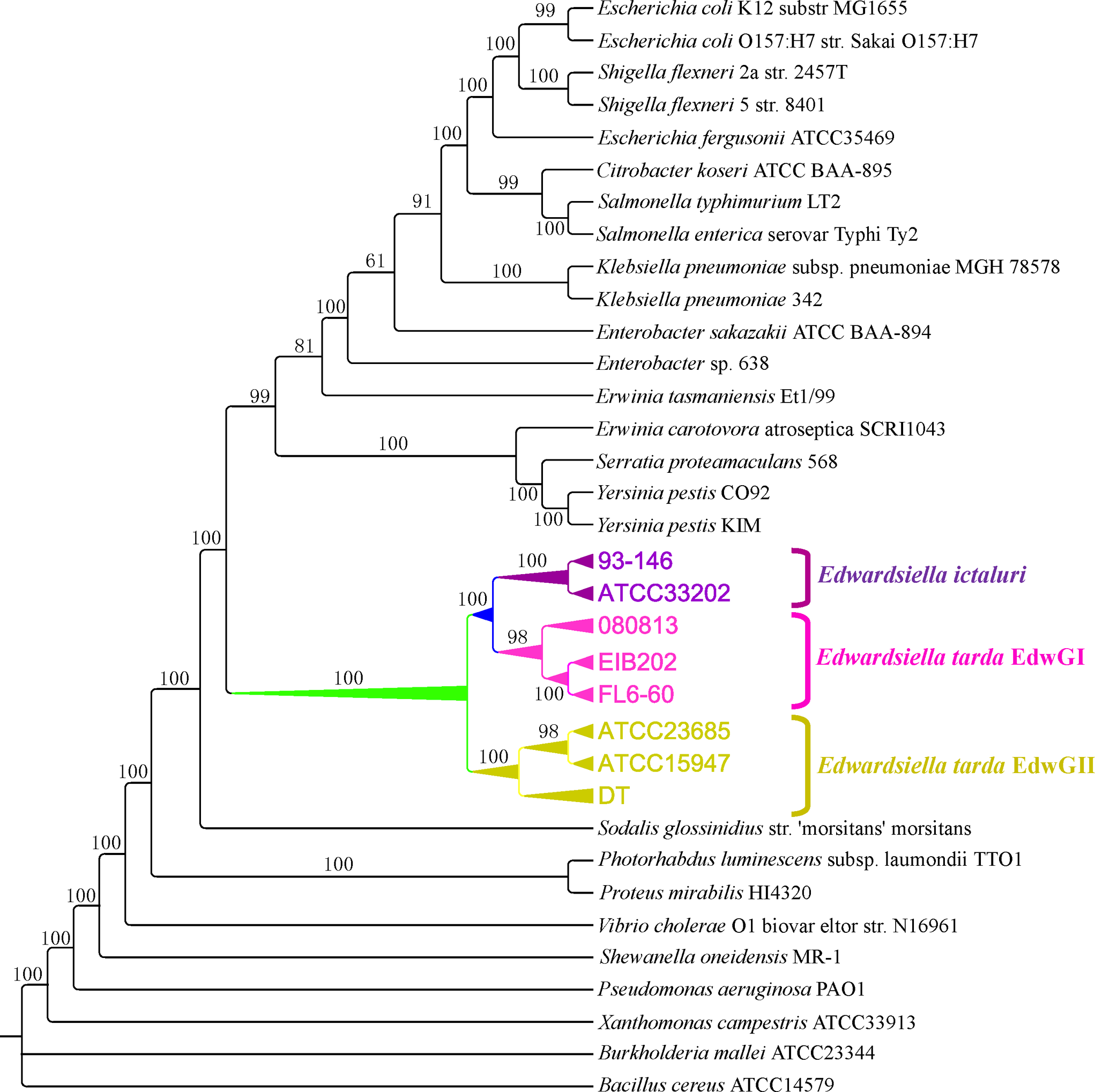

Supplement: Figure S1 — Phylogenetic tree of Edwardsiella species. Phylogenies of Edwardsiella species inferred from concatenated alignments of the protein sequences encoded by 44 house-keeping genes (adk, aroC, dnaA, dnaK, frr, fusA, gapA, gyrA, gryB, infC, nusA, pgk, phoB, phoR, pyrG, recC, rplA, rplB, rplC, rplD, rplE, rplF, rplK, rplL, rplM, rplN, rplP, rplS, rplT, rpmA, rpoA, rpoB, rpoC, rpoE, rpsB, rpsC, rpsE, rpsI, rpsJ, rpsK, rpsM, rpsS, smpB, and tsf) by PhyML program with 100 bootstrap iterations for clade support. Bacillus cereus ATCC14579 was used as the outgroup strain. (TIF) [file pone.0036987.s001.tif]

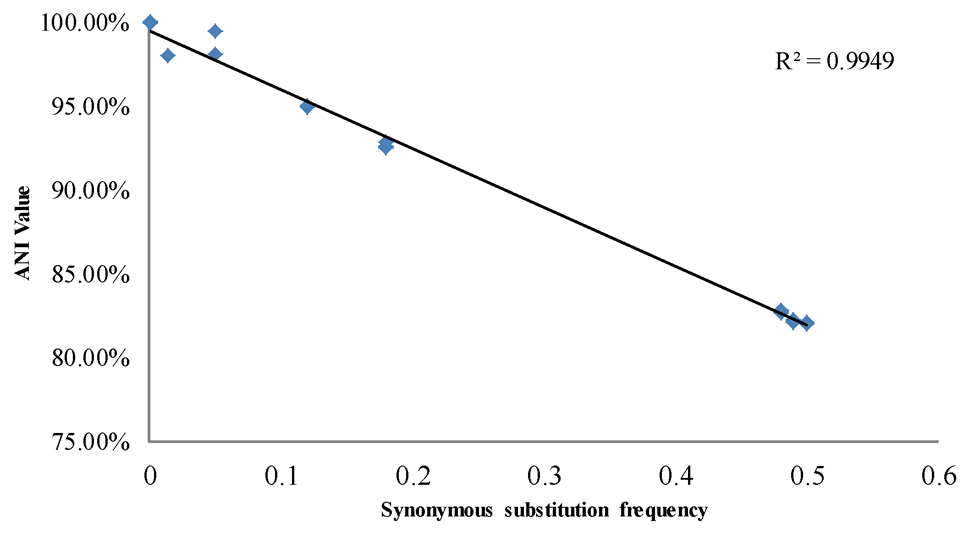

Supplement: Figure S2 — Relationships between ANI and synonymous nucleotide substitutions. Each blue square represents the ANI of all genome sequence between two strains (x axes) plotted against (y axes) the average rate of synonymous nucleotide substitutions of housekeeping genes. (TIF) [file pone.0036987.s002.tif]

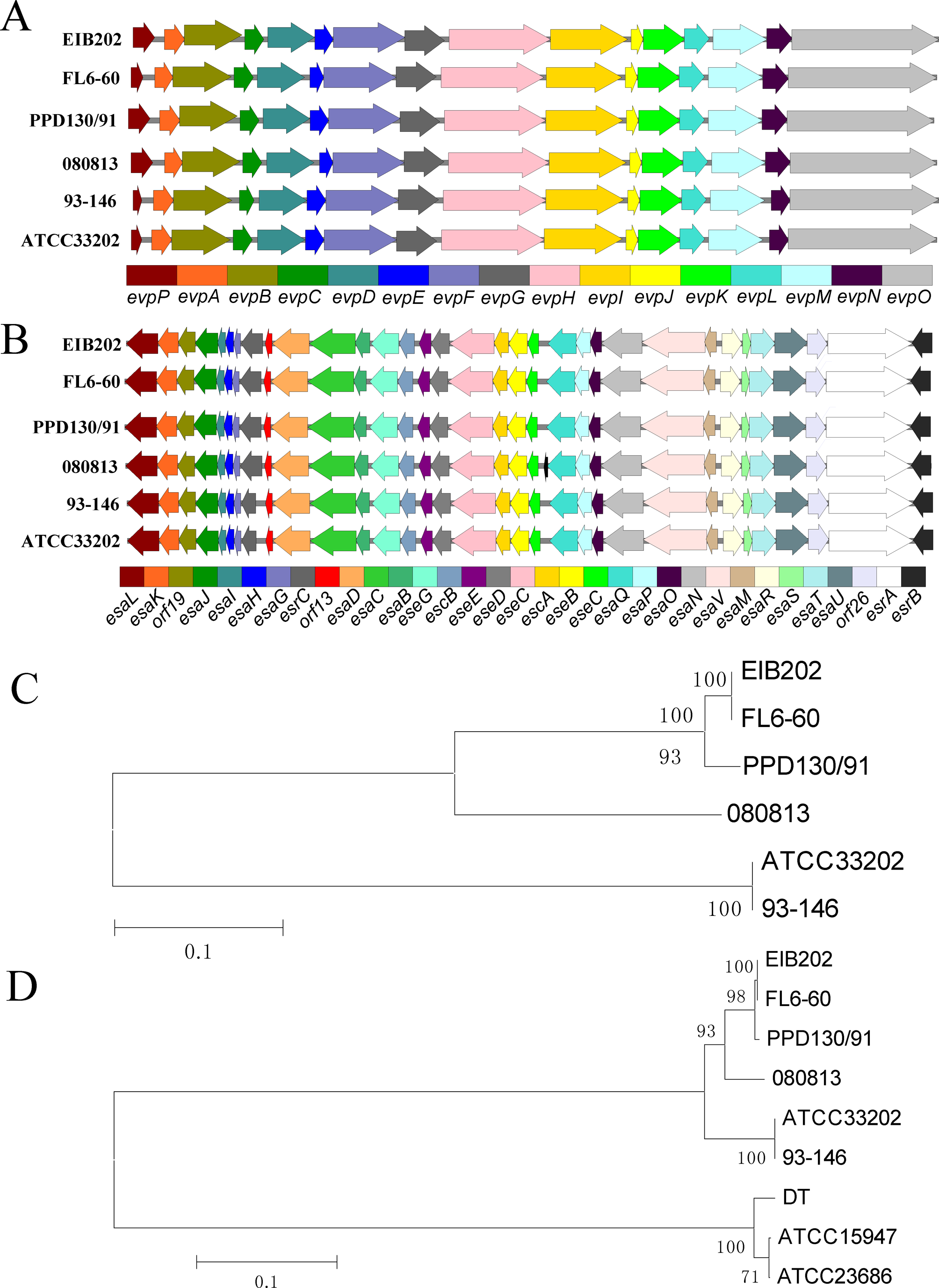

Supplement: Figure S3 — Edwardsiella virulence gene clusters of secreted proteins. T6SS (A) and T3SS (B) gene clusters of sequenced E. tarda EdwGI and E. ictaluri strains. All genes with high similarity are indicated in the same color and the gene names are shown below according to the color scheme. (C) NJ-tree of 6 Edwardsialla isolates (3 EdwGI strains, 2 E. icatluri strains and E. tarda PPD130/91) inferred from concatenated T6SS and T3SS aligned sequences. (D) NJ tree of 8 Edwardsiella species inferred from concatenated alignments of the coding sequences of esrA and esrB genes with 1000 bootstrap iterations. (TIF) [file pone.0036987.s003.tif]

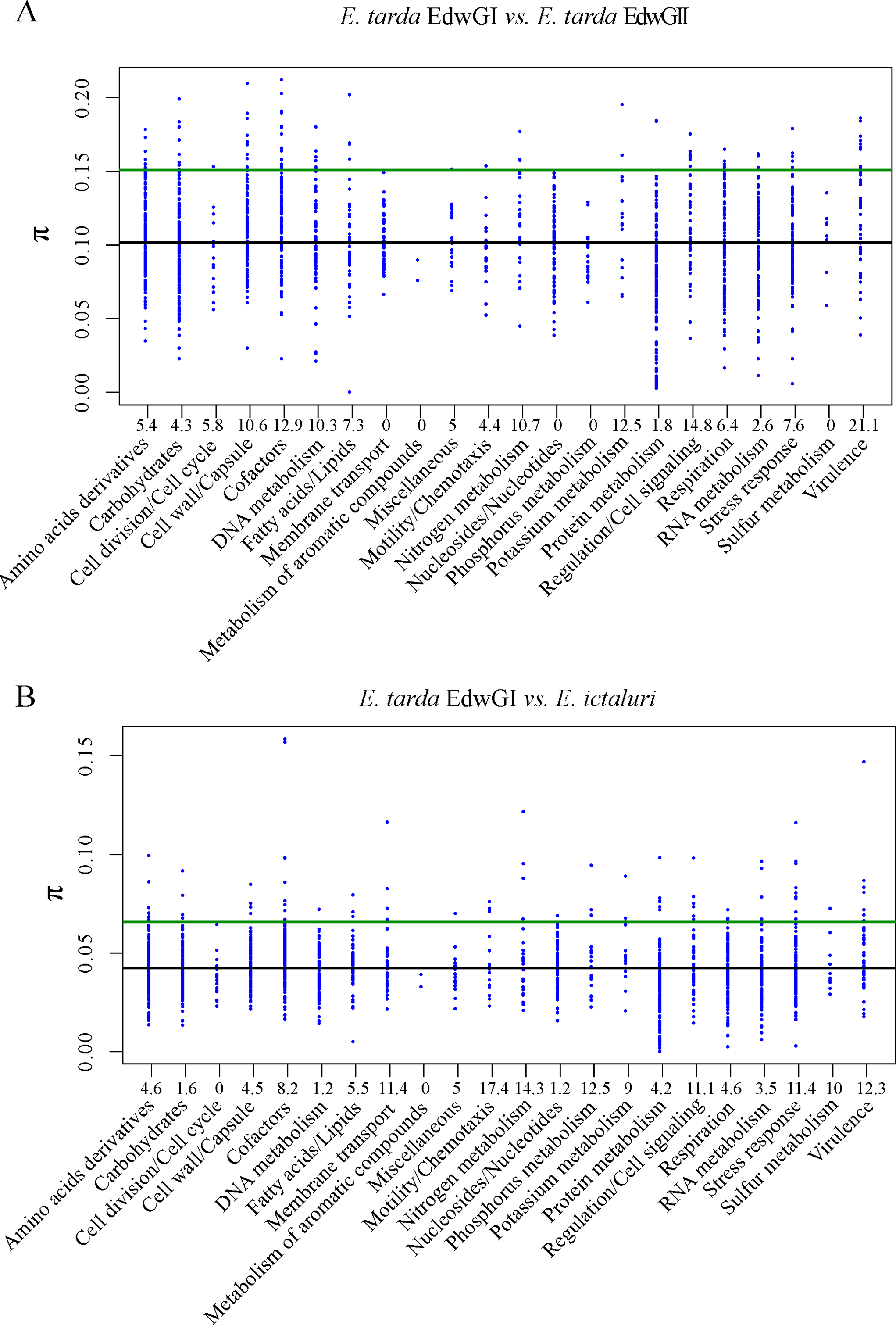

Supplement: Figure S4 — Nucleotide diversity (π) of orthoglous of Edwardsiella . (A) Nucleotide diversity (π) for E. tarda EdwGI and EdwGII. (B) Nucleotide diversity (π) for E. tarda EdwGI and E. ictaluri strains. The blank line represents the average π value of all orthologs. Green line indicates π values above 1.5σ (standard deviation) from the average π values of all orthologs, respectively. The percent of genes with π values large than 1.5σ from the average π value in each function category are shown under x axis. (TIF) [file pone.0036987.s004.tif]
