# Supplementary material for: Edwardsiella Comparative Phylogenomics Reveal the New Intra/Inter-Species Taxonomic Relationships, Virulence Evolution and Niche Adaptation Mechanisms
Source: PLoS One. 2012 May 10;7(5):e36987. doi: 10.1371/journal.pone.0036987 (PMC3349661; doi:10.1371/journal.pone.0036987)
Supplement: Table S1 — API-20E test of Edwardsiella strains. (DOC) [file pone.0036987.s005.doc]

**Table S1 Biochemical characteristics of the strains with API test strips**

|  | 080813 | DT | EIB202 | ATCC15947 | ATCC33202 |
| --- | --- | --- | --- | --- | --- |
| ONPG | - | - | - | - | - |
| ADH | - | - | - | - | - |
| LDC | + | + | + | + | + |
| ODC | + | + | + | + | - |
| CIT | + | + | - | - | - |
| H2S | + | + | + | + | - |
| URE | - | - | - | - | - |
| TDA | - | - | - | - | - |
| IND | + | + | + | + | - |
| VP | - | - | - | - | - |
| GEL | - | - | - | - | - |
| GLU | + | + | + | + | + |
| MAN | - | - | - | - | - |
| INO | - | - | - | - | - |
| SOR | - | - | - | - | - |
| RHA | - | - | - | - | - |
| SAC | - | - | - | - | - |
| MEL | - | - | - | - | - |
| AMY | - | - | - | - | - |
| ARA | - | - | - | - | - |

API 20E test of *Edwardsiella* strains. All the data were collected after 16 h incubation at 37°C. ONPG, β-galactosidase; ADH, arginine dihydrolase; LDC, lysine decarboxylase; ODC, ornithine decarboxilase; CIT, citrate utilization; H2S, H_2_S production; URE, urease; TDA, tryptophane deaminase; IND, indole production; VP, Voges–Proskauer; GEL, gelatinase; GLU, glucose; MAN, mannitol; INO: inositol; SOR: sobitol; RHA, rhamnose; SAC; saccharose; MEL, melibiose; AMY: amygdalin; ARA, arabinose.
