# Supplementary material for: Edwardsiella Comparative Phylogenomics Reveal the New Intra/Inter-Species Taxonomic Relationships, Virulence Evolution and Niche Adaptation Mechanisms
Source: PLoS One. 2012 May 10;7(5):e36987. doi: 10.1371/journal.pone.0036987 (PMC3349661; doi:10.1371/journal.pone.0036987)
Supplement: Table S3 — Sequence identity of 8 Edwardsiella strains. (DOC) [file pone.0036987.s007.doc]

Table S3. Average identity rate between sequenced strains

|  | **EIB202** | **FL6-60** | **080813** | **93-146** | **ATCC33202** | **ATCC15947** | **DT** | **ATCC23685** |
| --- | --- | --- | --- | --- | --- | --- | --- | --- |
| EIB202 | - | 1.08*E*-01 | 4.38*E*-01 | 0.00*E*+00 | 0.00*E*+00 | 0.00*E*+00 | 0.00*E*+00 | 0.00*E*+00 |
| FL6-60 | 98.03 | - | 4.38*E*-01 | 0.00*E*+00 | 0.00*E*+00 | 0.00*E*+00 | 0.00*E*+00 | 0.00*E*+00 |
| 080813 | 94.00 | 94.12 | - | 1.80*E*-06 | 5.20*E*-06 | 0.00*E*+00 | 0.00*E*+00 | 0.00*E*+00 |
| 93-146 | 92.24 | 93.01 | 92.01 | - | 1.00*E*+00 | 0.00*E*+00 | 0.00*E*+00 | 0.00*E*+00 |
| ATCC33202 | 92.24 | 92.09 | 92.23 | 97.15 | - | 0.00*E*+00 | 0.00*E*+00 | 0.00*E*+00 |
| ATCC15947 | 87.09 | 85.79 | 86.46 | 85.80 | 85.78 | - | 7.22*E*-03 | 7.22*E*-03 |
| DT | 85.87 | 86.46 | 86.76 | 86.55 | 86.70 | 96.93 | - | 7.22*E*-03 |
| ATCC23685 | 86.65 | 86.91 | 87.64 | 87.16 | 87.50 | 98.74 | 96.91 | - |

MuMmer nucmer was used for sequence comparison with default parameters and the output between query and target genomes was used for one-way ANOVA analysis and Tukey's HSD test. Average sequence identity between EIB202 and other *Edwardsiella* strains (Left) and adjusted *p* value of Tukey's HSD test of all genome sequences between two strains (Right) are shown.
