# Supplementary material for: Edwardsiella Comparative Phylogenomics Reveal the New Intra/Inter-Species Taxonomic Relationships, Virulence Evolution and Niche Adaptation Mechanisms
Source: PLoS One. 2012 May 10;7(5):e36987. doi: 10.1371/journal.pone.0036987 (PMC3349661; doi:10.1371/journal.pone.0036987)
Supplement: Table S6 — ANI value and synonymous substitution frequency of the strains studied. (DOC) [file pone.0036987.s010.doc]

**Table S6. ANI value and average synonymous substitution frequency between two strains**

|  | EIB202 | FL6-60 | 080813 | 93-146 | ATCC  33202 | ATCC  15947 | ATCC  23685 | DT |
| --- | --- | --- | --- | --- | --- | --- | --- | --- |
| EIB202 | - | 99.93 | 94.92 | 92.53 | 92.52 | 82.62 | 82.8 | 82.69 |
| FL6-60 | 0.0004±0.007 | - | 94.99 | 92.57 | 92.54 | 82.62 | 82.8 | 82.64 |
| 080813 | 0.12±0.05 | 0.12±0.05 | - | 92.85 | 92.85 | 82.74 | 82.83 | 82.71 |
| 93-146 | 0.18±0.07 | 0.18±0.7 | 0.18±0.08 | - | 99.98 | 82.07 | 82.24 | 82.15 |
| ATCC33202 | 0.18±0.07 | 0.18±0.07 | 0.18±0.08 | 0.0005±0.008 | - | 81.96 | 82.04 | 81.96 |
| ATCC15947 | 0.48±0.14 | 0.48±0.14 | 0.48±0.14 | 0.49±0.14 | 0.50±0.14 | - | 99.43 | 98.09 |
| ATCC23685 | 0.48±0.14 | 0.48±0.14 | 0.48±0.14 | 0.49±0.14 | 0.50±0.14 | 0.014±0.021 | - | 98 |
| DT | 0.48±0.14 | 0.48±0.14 | 0.48±0.14 | 0.49±0.14 | 0.50±0.14 | 0.05±0.03 | 0.05±0.03 | - |

Average synonymous substitution frequency (Ds) values from 1000 house-keeping orthologous gene pairs from the core gene set (COG C, D, E, F, G, I, J, K, L and P) according to the method of Nei and Gojobori [1](Left) and ANI vaule of all genome sequence between two strains [2,3] (Right).

1. Nei M, Gojobori T (1986) Simple methods for estimating the numbers of synonymous and nonsynonymous nucleotide substitutions. Molecular biology and evolution 3: 418-426.

2. Konstantinidis KT, Tiedje JM (2005) Genomic insights that advance the species definition for prokaryotes. Proceedings of the National Academy of Sciences of the United States of America 102: 2567.

3. Goris J, Konstantinidis KT, Klappenbach JA, Coenye T, Vandamme P, et al. (2007) DNA-DNA hybridization values and their relationship to whole-genome sequence similarities. International journal of systematic and evolutionary microbiology 57: 81-91.
